# Clients' experiences of psychotherapeutic interventions addressing trauma

**DOI:** 10.1111/papt.12569

**Published:** 2025-01-03

**Authors:** R. Lepistö, A. Ahmad, S. Kangaslampi, K. Peltonen

**Affiliations:** ^1^ Faculty of Social Sciences/Psychology Tampere University Tampere Finland; ^2^ INVEST Flagship Research Centre University of Turku Turku Finland

**Keywords:** client experience, qualitative, systematic review, thematic synthesis, trauma

## Abstract

**Purpose:**

This systematic review aimed to evaluate and synthesise qualitative research on adult clients' experiences of psychotherapeutic interventions addressing trauma across multiple modalities.

**Methods:**

Six databases (PsycINFO, MEDLINE, The Cochrane Central Register of Controlled Trials, Web of Science, Scopus, and CINAHL) were systematically searched. Google Scholar and reference lists of included and other relevant reviews were also searched, and in total 37 studies met the inclusion criteria. Relevant data were extracted, quality assessed following the Critical Appraisal Skill Programme checklist, and data synthesised using thematic synthesis.

**Results:**

The specific helpful factors and perceived impact of the interventions aligned with their theoretical bases. Certain factors were perceived to be helpful or challenging regardless of the modality used, such as relational factors between the client and the therapist, and within group settings. Towards the end of the interventions, feelings of ambivalence and emotional struggles which pervaded the process gradually resolved, as a majority of the clients expressed a sense of benefit from the whole experience. Most of the studies included in this review were published post‐2020, underscoring research interest towards clients' psychotherapeutic experiences.

**Conclusions:**

The review provides a comprehensive understanding of helpful and challenging factors of interventions addressing trauma, as perceived by clients. The analysis serves as a foundation for future intervention development. Also, it highlights the importance of therapist responsiveness and discussions with clients at various intervention stages to foster a sense of safety, prevent early drop‐outs, and emphasise clients as agents of change in their therapeutic process.

## INTRODUCTION

Over the past three decades, research in the field of traumatic stress has advanced singnificantly, reflecting the high prevalence of trauma exposure, which is estimated to affect approximately 70% of the global population during their lifetime (Kessler et al., 2017). Following trauma exposure, many individuals experience posttraumatic symptoms, including intrusive thoughts, avoidance behaviours, and hypervigilance, which are characteristics of posttraumatic stress disorder (PTSD). The 11th edition of the International Classification of Diseases (ICD‐11) (World Health Organization, 2019) introduced two closely related disorders: PTSD, a prevalent, debilitating, and often chronic condition, and Complex Posttraumatic Stress Disorder (CPTSD), which is marked by symptoms of PTSD along with negative self‐belief, emotional dysregulation, and relational difficulties. Through this extensive research, psychotherapeutic interventions have been developed and validated across diverse populations, settings, and trauma types (e.g. Cusack et al., 2016).

Various psychotherapeutic interventions have been developed to address trauma‐specific issues, encompassing diverse theoretical frameworks. Meta‐analyses and systematic reviews (e.g., Cusack et al., 2016; Watts et al., 2013) indicate that trauma‐focused interventions such as cognitive‐behavioural and cognitive therapy approaches and Eye Movement Desensitisation and Reprocessing (EMDR), which include elements such as revisiting the traumatic memory, exhibit the most substantial evidence for effectiveness and are first‐line recommendations in PTSD treatment guidelines (APA, 2017). However, despite the recognised significance of common factors, such as a good therapeutic alliance between the therapist and the client, in contributing to the effectiveness of psychotherapy, such elements are notably absent from these treatment recommendations (Norcross & Wampold, 2019). Previous literature has emphasised the importance of good therapeutic alliance, such as its impact on participant dropout (Sijercic et al., 2021) and improving participants' ability to regulate negative mood during treatment (Cloitre et al., 2004).

Numerous researchers advocate a deeper understanding of psychotherapy through a focus on qualitative evidence derived from clients' own experiences, underscoring the pivotal role of clients in the therapeutic process (Bohart & Wade, 2013; Elliott, 2008; Fuertes & Williams, 2017; Hodgetts & Wright, 2007; Levitt et al., 2016). Qualitative approaches, such as investigating helpful and hindering aspects of the psychotherapy process, offer a valuable lens into clients' experiences. Timulak and Keogh (2017) highlighted a common thread across these studies regarding the value clients place on a non‐judgemental, caring, and supportive therapeutic relationship where they feel understood and accepted. Consequently, qualitative information not only enhances therapists' sensitivity to clients' experiences but also provides detailed accounts of how interventions can impact clients. This wealth of information can aid in tailoring interventions and, further, in matching clients to suitable interventions. Moreover, the integration of client feedback during sessions, as seen in approaches like feedback‐informed treatment (Duraisamy & Baeder, 2015), facilitates ongoing tailoring of interventions.

Previous literature such as Parry and Simpson (2016) conducted a systematic review of the experiences of adult survivors of CSA in nonspecific and trauma‐focused talking therapies, comprising 23 qualitative studies. In addition, Whitehouse (2021) and Shipley et al. (2022) conducted a systematic review of clients' experiences of Eye Movement Desensitisation and Reprocessing (EMDR). Whitehouse's (2021) review incorporated five qualitative studies, while Shipley et al. (2022) additionally concentrated on clients' negative experiences and incorporated grey literature, including 13 studies. Moreover, Gjerstad et al. (2024) conducted a systematic review of experiences of trauma‐focused therapy (TFT) among adults with PTSD compromising 9 qualitative studies.

The widespread prevalence of trauma underscores the need for continual improvements in psychotherapeutic interventions. However, past reviews have primarily focused on single intervention modalities or one type of trauma (e.g., only including CSA in the review), lacking comprehensive comparisons across multiple approaches and types of trauma, whereas this review incorporates various psychotherapeutic interventions. Furthermore, qualitative research emerges as a valuable approach for assessing the effectiveness of psychotherapeutic interventions, as it reveals nuanced and in‐depth insights. By focusing on clients' experiences within these interventions, this review aims to uncover the various factors contributing to their effectiveness. Through a systematic analysis, it seeks to provide a comprehensive understanding of the distinctions and commonalities in clients' experiences of different psychotherapeutic modalities addressing trauma which have not been addressed previously. This study defines psychotherapeutic intervention as a systematic, goal‐directed, language‐driven interaction, guided by health care principles and ethical standards, rooted in comprehensive theoretical knowledge. It involves an interaction between a client and a therapist, encompassing both individual and group contexts, with the explicit aim of addressing mental health issues arising from exposure to traumatic event(s).

## RATIONALE AND AIMS OF THE PRESENT STUDY

This systematic review aimed to evaluate and synthesise research findings of adult clients' experiences with psychotherapeutic interventions addressing trauma, focusing on helpful and challenging factors during the intervention process and the impacts following its conclusion, to provide a comprehensive picture of the phenomenon. It aimed to facilitate understanding of clients' experiences of psychotherapeutic interventions addressing trauma across multiple modalities with systematic and rigorous methodology (Timulak, 2009). The review questions were: (1) What are clients' experiences of helpful and challenging factors in psychotherapeutic interventions addressing trauma? and (2) How do clients perceive the impact of the psychotherapeutic interventions addressing trauma? As the research questions highlight, this review includes multiple psychotherapeutic modalities to explore the general experiences of clients, rather than focusing on a specific approach, which previous studies have extensively concentrated on. In contrast, this review offers a broad overview of client experiences across different interventions. By highlighting commonalities among various modalities, it lays the groundwork for future research and serves as a comprehensive reference for practitioners in intervention planning.

### Method

This systematic review encompassed all published qualitative studies (without publication date restrictions) on clients' experiences with psychotherapeutic interventions addressing trauma. It aimed to produce a data‐driven thematic synthesis of primary findings. Qualitative components of mixed‐method studies that met the research question requirements and inclusion criteria were also included.

### Protocol and registration

The protocol of this systematic review was registered in the PROSPERO database [https://www.crd.york.ac.uk/prospero/display_record.php?RecordID=340008].

### Search strategy

After scoping searches and consulting an information specialist, the first author conducted a systematic search in PsycINFO, Scopus, and CINAHL and the second author in MEDLINE, Web of Science, and the Cochrane Central Register of Controlled Trials. Searches were conducted in June 2022 and again in October 2023. The first author also hand‐searched other sources such as Google Scholar and reviewed references of included studies and other relevant reviews.

The Sample, Phenomenon of Interest, Design, Evaluation and Research type (SPIDER) tool was used to structure the eligibility criteria and search strategy (Cooke et al., 2012); search terms were adjusted to each database's practices and included terms related to the client (S), trauma and intervention modality (P, I), qualitative study design (D), experience (E), and qualitative research type (R). A detailed description of the search terms is presented in Table 1. An example of a search string is presented in Supplementary Material.

**TABLE 1:** Literature search strategy.

Search category	Search terms
Sample (S)	adult* or client* or participant* or patient* or user*
Phenomenon of interest (P, I)	trauma* or ptsd or ptss or post‐traumatic stress* and psychological treatment* or therapy* or psychotherapy* or intervention* or counseling* or behavioral* or brief eclectic* or client‐centered* or cognitive* or cognitive‐analytic* or cognitive processing* or dialectical behavioral* or ego‐state* or emdr or eye movement desensitization and reprocessing* or emotion‐focused* or feminist therapy* or gestalt* or humanistic* or hypno* or integrative* or internal family systems* or interpersonal* or narrative exposure therapy* or person‐centered* or prolonged exposure* or psychoanalytic* or psychoanalysis* or psychodynamic* or sensorimotor* or solution‐focused* or somatic experiencing* or trauma‐focused cognitive behavioral therapy* or tf‐cbt
Design (D)	case stud* or consensual qualitative* or content analy* or discourse analysis* or ethnographic* or field study* or focus group* or grounded theory* or interview* or lived experience* or narrative* or observ* or phenomenological* or process evalua* or significant moments* or survey* or thematic analy*
Evaluation (E)	attitude* or experienc* or opinion* or perce* or response* or view*
Research type (R)	qualitative* or mixed‐meth* or mixed meth* or multi‐meth* or multi meth*

*Note*: Sample (S) was not in the key strings to avoid search string complications.

### Screening and selection criteria

Initially, titles and abstracts were screened through different databases by the first and second authors independently, using predefined eligibility criteria (as described below). Both authors selected relevant studies and transferred them into a reference management programme (Rayyan; Ouzzani et al., 2016), where duplicate studies were removed. The first and second authors individually retrieved the remaining studies for full‐text review. The final list of articles was determined through multiple discussions between the two authors, ensuring that each article thoroughly addressed the research questions. The third and fourth authors were consulted to review articles that included add‐on interventions and only partially addressed the research questions. This resulted in revising the inclusion criteria such as the exclusion of add‐on interventions and case studies.

### Inclusion criteria

Studies were included if (A) the research questions were fully addressed. (B) A qualitative or mixed‐methods design (of which only the qualitative part) was used to investigate and report findings of multiple clients' experiences of the intervention from interviews or focus groups. Case studies were excluded as distinguishing specific client experiences from compiled results had the possibility of a high error margin. (C) Participants were current or former adult (18+) clients of a psychotherapeutic intervention addressing trauma. (D) For the review, participants had been exposed to a traumatic event(s) according to the ICD‐11 criterion, i.e., an event or situation (either short‐ or long‐lasting) of an extremely threatening or horrific nature. The ICD‐11 criterion was selected as it has a higher user prevalence (Cao et al., 2020). (E) Studies reported either the traumatic event(s) or symptoms/diagnosis as a consequence of trauma exposure. Studies were included if 50% of the participants were exposed to traumatic event(s) fulfilling the ICD‐11 criterion. (F) The studied interventions were psychotherapeutic, defined as being based on the same principles as psychotherapy (as described earlier). (G) Interventions explicitly treated consequences of trauma exposure. (H) Studies reported experiences of the main psychotherapeutic intervention or the factors generally belonging to it. Studies reporting experiences of only add‐ons (e.g., somatic techniques such as massages) were excluded. (I) Intervention could be in‐person with a therapist or therapeutic group or conducted remotely with a telehealth therapist. Studies in which psychoeducation was the only modality were excluded. (J) Studies were published in peer‐reviewed journals and reported in English.

### Quality appraisal, data extraction, and synthesis

The quality of each article was assessed by the first and second authors independently following the Critical Appraisal Skill Programme (CASP) tool (2018), which is a widely used system for this purpose. It included questions such as ‘Was there a clear statement of the aims of the research?’ and ‘Is a qualitative methodology appropriate?’ (see Table 3). The quality appraisal tool will be discussed in the results section.

Based on the research questions, key information was extracted from each article and set out in an Excel spreadsheet by the first author and checked by the other authors. This included information such as the intervention modalities, number of sessions, and country in which the intervention took place (see Table 2). Information regarding the characteristics of the participants is mentioned under ‘Characteristics of Included Studies’.

**TABLE 2:** Characteristics of included articles.

Article	Intervention modality	Context, country	Sessions	Data collection	Data analysis method	Sample size
Ashfield et al. (2021)	CFT, Compassionate‐Resilience^†^	Specialist PTSD service, UK	Unspecified	After completion, unspecified	Constructivist GT, SSI	11
Bahu (2019)	Culturally adapted CBT^†^	Improving Access to Psychological Therapies service, UK	12 × 2 h over 3 months	After completion, unspecified	TA, verbal feedback	16
Bragesjö et al. (2021)	Internet‐delivered PE*	Unspecified, Sweden	3 weeks, 4 modules	6 months after completion	TA, SSI	11
Chouliara et al. (2020)	TREM^†^	Human service agencies, Scotland, UK	29 × 75 min	After completion, unspecified	IPA, SSI	16
de Haan et al. (2021)	Imagery rescripting*	Several sites, Australia, Germany, Netherlands	12 × 90 min, twice weekly for 6–8 weeks	After completion, unspecified	TA, SSI	44
Edmond et al. (2004)	EMDR and Eclectic therapy*	Unspecified, US	6 × 90 min	After completion, unspecified	Structured five‐stage analysis process of SSI	38
Forde and Duvvury (2021)	Humanistic integrative psychotherapeutic approach*	Rape crisis centre, Ireland	<1 year to 3–5 years	After completion, unspecified	TA, SSI	11
Gnall et al. (2020)	Cognitive‐behavioural trauma‐informed intervention^†^	Veteran affairs services, USA	12 weeks	After final session	TA of post‐treatment satisfaction responses	291
Hundt et al. (2017)	PE, CPT^*†^	Veteran Affairs PTSD clinic, USA	8–15	After completion, unspecified	GT, SSI	23
Hundt et al. (2020)	PE and CPT*	Veteran's Health Administration PTSD clinic, USA	At least 1 but <8	After dropping out, unspecified	GT, SSI	28
Joubert and Guse (2022)	SFBT*	Public mental health services, South Africa	1 to 4 times 60mins	1–2 weeks after completion	TA, SSI and audio‐recorded therapy sessions	7
Kehle‐Forbes et al. (2022)	PE, CPT*	Veteran affairs clinic, USA	Non‐completers 1–6 sessions of PE/CPT; completers at least 10 PE or 12 CPT sessions	After completion or dropping out, unspecified	Qualitative analysis, SSI	126
König et al. (2020)	DET, CBT*	University clinic, Germany	Minimum 24 weekly sessions	After each session	Qualitative content analysis	110
Lawrence and Lee (2014)	CFT^*†^	Unspecified, UK	Unspecified	After completion, unspecified	IPA, SSI	7
Lowe and Murray (2014)	TF‐CBT*	Specialist PTSD outpatient treatment service, UK	Average 12	1–4 weeks post intervention	IPA, interviews	9
Matheson and Weightman (2021)	Female group, NET, EMDR, Systemic therapy, Male group, TF‐CBT, Psychodynamic^*†^	Trauma service, UK	12–74, average 36.5	Over 12 weeks after completion	Participatory approach, interviews	24
May et al. (2022)	EMDR, CFT, Integrative trauma‐focused therapy*	Unspecified, UK	Unspecified	Within 12 months of finishing treatment	IPA, SSI (interviews via telephone)	9
McGregor et al. (2006)	Not specified	Unspecified, New Zealand	At least 5	After completion, unspecified	GT, SSI	20
Middle and Kennerley (2001)	Not specified	Unspecified, UK	At least 6	After completion, not specified	GT, SSI	17
Mirdal et al. (2012)	Short‐term psychodynamic therapy	Rehabilitation for traumatised refugees, Denmark	31–41	6–20 months after completion	Qualitative phenomenological approach, SSI	16
Mott et al. (2013)	Exposure therapy^†^	Veterans Affairs PTSD speciality clinic, USA	3 hrs twice a week for 12 weeks	About 6 months after completion	Analytic approach method	20
Murray et al. (2016)	TF‐CBT*	Specialist outpatient service for PTSD, UK	Conducted over 2 years, Unspecified	After site visit during treatment	GT, free‐text written responses	25
Røberg et al. (2018)	Trauma Stabilising group^†^	Outpatient clinic, Norway	22 × 2 h weekly	After completion, unspecified	IPA, SSI	5
Schwarz et al. (2020)	EMDR*	Underserved community agency, USA	8 sessions	After completion, unspecified	Unspecified qualitative analysis, SSI	15
Schwarz et al. (2021)	EMDR*	Non‐profit counselling agency, USA	8 sessions	After completion, unspecified	Constant comparative method, SSI	21
Shearing et al. (2011)	TF‐CBT*	Specialist trauma service, UK	Unspecified	After one reliving component completed	IPA, SSI	7
Sherrill et al. (2022)	Massed PE^*†^	Multiple veteran and community clinics, USA	2‐weeks, 9× daily 90 min individual sessions, 9× daily 120 min group sessions	After final treatment session	TA, open‐ended survey responses	25
Stige, Rosenvinge, et al. (2013), Stige, Binder, et al. (2013) and Stige et al. (2019)	Phase‐oriented stabilisation group ^†^	Outpatient setting of specialised mental health services, Norway	17 × 90 min weekly	Immediately after completion and 1 year after completion	Hermeneutic‐phenomenological approach, SSI	13
Thoresen et al. (2022)	Intensive PE, EMDR	Outpatient clinic specialising in severe post‐traumatic stress, Norway	4 days per week over a 2‐week period with 90 min PE and EMDR (each)	2 weeks after intervention	TA, SSI	8
Valentine and Smith (1998)	Traumatic Incident Reduction*	Unspecified, US	At least 1 session	After completion, exit interviews	Ethnoscience, SSI	16
Vincent et al. (2013)	TF‐CBT*	Outpatient services specialising in PTSD, UK	2–10, average 3	After minimum 2 sessions of TF‐CBT	IPA, SSI	7
Visagie and Keet (2021)	Eye movement integration*	Unspecified, South Africa	Unspecified	After completion, unspecified	Exploratory‐descriptive research design, SSI	10
Vlasova (2017)	Systemic Psychotherapy*	Psychotherapeutic clinic for refugees and asylum seekers, Austria	10–39 over 6–12 months	Unspecified	GT, SSI, and recorded therapy sessions	10
Wästlund et al. (2023)	TMC^†^	Specialised outpatient facility for CPTSD, Norway	16 × 2 h weekly	Within 4 weeks of completion	Reflective TA, SSI	17
Younan et al. (2018)	Schema therapy^*†^	Private psychiatric hospital, Australia	4 weeks	Pre‐, post‐ and 3 months after intervention	TA, SSI	12

*Note*: ^†^Group sessions, *Individual sessions.

Abbreviations: (TF‐)CBT, Trauma‐Focused Cognitive Behavioural Therapy; CFT, Compassion‐focused Therapy; CPT, Cognitive Processing Therapy; EMDR, Eye Movement Desensitisation Reprocessing; GT, Grounded Theory; IPA, Interpretative Phenomenological Analysis; NET, Narrative Exposure Therapy; PE, Prolonged Exposure; SFBT, Solution‐Focused Brief Therapy; SSI, Semi‐structured interviews; TA, Thematic Analysis; TMC, Trauma‐sensitive Mindfulness and Compassion; TREM, Trauma Recovery and Empowerment Model.

For analysis purposes, all selected studies were entered into ATLAS.ti (23.1.1.0 for Windows, 2023) software. A thematic synthesis, commonly used to analyse multiple qualitative studies, was employed for this review. The process provides an overarching interpretation of the data by following three main stages (as described by Thomas & Harden, 2008).

*Stage 1* involves line‐by‐line coding of the primary texts, conducted by the first author. After generating codes, both the first and second authors critiqued their comprehension; examined similarities, overlaps, and differences; and produced a final list of codes. This collaborative process continued until both authors agreed on the representability of the codes.

*Stage 2* focuses on the development of descriptive themes. This was achieved by grouping similar codes that reflected the content of the dataset—specifically, the results sections of the included studies. The discussion sections were not coded to avoid relying on the interpretations of the study authors. The grouped codes were assigned comprehensive titles, which became the basis for second‐level themes. These titles were continuously reviewed and refined to ensure they were representative and specific to the descriptive subthemes.

*Stage 3* involves the development of analytical themes, labelled in this review as “superordinate themes”. These themes reflected the research questions: the first four superordinate themes address the first research question regarding clients' experiences of helpful and challenging factors of the interventions, while the last two superordinate themes address the second research question about the perceived impact of the interventions.

### Ethical considerations and reflexivity

No ethical approval was required due to the systematic review methodology. All authors are psychologists and researchers. The first two authors are PhD researchers and psychologists (Integrative psychotherapy student and EMDR practitioner, respectively). None of the authors have been part of the included studies. All authors are actively involved in continued professional development including learning about client‐based nuances of different therapeutic modalities, while being vigilant of their potential confirmation biases during the data analysis and synthesis process of the review. The authors held multiple discussions to reflect on their views and beliefs throughout the data gathering, analysis, and results presentation stages (especially between the first two authors), as well as to refresh and gain new perspectives (among all authors).

## RESULTS

### Identification of relevant studies

**FIGURE 1:**
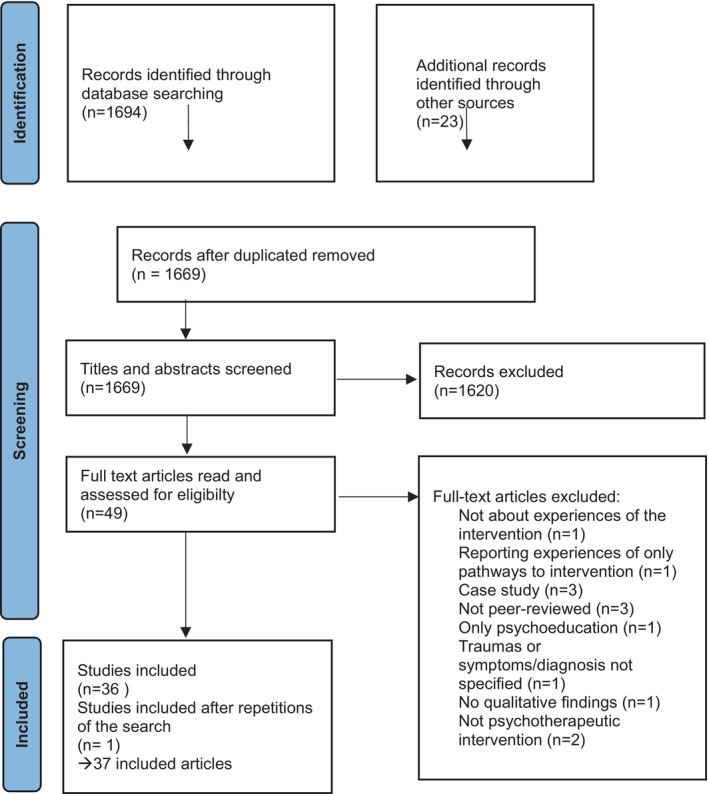
PRISMA flow diagram of selection of studies.

The initial search identified 1694 studies, and 23 studies were found from other sources as previously described. A total of 49 articles were selected for full‐text reading out of which 36 articles met the inclusion criteria (Stige, Binder, et al., 2013, Stige, Rosenvinge, et al., 2013; Stige et al., 2019 counted as one article in the analysis due to the same sample). One new article was added after repeating searches. The details of the search and screening processes are provided in the Preferred Reporting Items for Systematic Review and Meta‐Analyses (PRISMA) flow diagram (Moher et al., 2015) in Figure 1.

### Characteristics of included studies

Study characteristics are presented in Table 2. The main characteristics of the 37 studies included a range of intervention modalities within individual and group contexts. Eight studies included multiple modalities, and in two studies modalities were not specified (i.e., McGregor et al., 2006; Middle & Kennerley, 2001). One study was in online format (Bragesjö et al., 2021), while the others were face‐to‐face. The duration of interventions varied widely, ranging from a few weeks to several months. Five articles did not specify the number of sessions or the length of the intervention. One study extended from over a year to 3–5 years (Forde & Duvvury, 2021), another lasted between six to 12 months including 10–39 sessions (Vlasova, 2017), and a third had between 12 and 74 sessions, with an average of 36.5 sessions (Matheson & Weightman, 2021).

The studies included the views of a total of 1045 participants (*n* = 509 females; three articles only had male participants, i.e., Gnall et al., 2020, 291 males; Mott et al., 2013, 20 males; and Røberg et al., 2018, 5 males) and the age range was 18–78 years. Traumatic events varied from single incidents to prolonged, repeated traumas. These included but were not limited to physical, emotional, and sexual abuse, childhood and domestic violence, and war trauma (such as witnessing torture and imprisonment). Furthermore, studies included people of various ethnicities such as individuals from Australasia, Europe (including Hispanics), Scandinavian countries, South Africa, and the United States. One study (Bahu, 2019) was conducted in the United Kingdom, but all participants were Nepali asylum‐seeking individuals.

Most studies were based in the United Kingdom (*n* = 11) and the United States (*n* = 10) and were carried out in specialised clinics for PTSD or trauma (*n* = 14) and Veterans Affairs centres (*n* = 4). Out of the 37 studies, three Norwegian studies (Stige, Binder, et al., 2013; Stige, Rosenvinge, et al., 2013; Stige et al., 2019) were conducted with the same participants and are presented in Table 2 as one study. All included studies were published from 1998 to 2023, except four articles (i.e., Edmond et al., 2004; McGregor et al., 2006; Middle & Kennerley, 2001 and Valentine & Smith, 1998); the rest were published from 2010 onwards, including 18 studies from 2020 onwards.

Data on the clients' experiences were collected at varying time points. In most studies, data was collected in the final session or up to 1 year after intervention. Only one study collected data during the intervention (i.e., König et al., 2020). In 15 studies, the trauma diagnosis and consequences were measured with the help of questionnaires and/or structured clinical interviews. The remaining studies based diagnoses and symptoms on unreported assessments or diagnoses established in previous interventions. Some specialised clinics for veterans and rape survivors did not mention diagnosis measurements and in one article (i.e., Valentine & Smith, 1998) trauma was defined by clients.

Of the 37 studies, 30 were qualitative and seven employed mixed‐method design; the most commonly used methods were thematic analysis (*n* = 10), grounded theory (*n* = 7), and interpretative phenomenological analysis (*n* = 6) of semi‐structured interviews.

### Quality of included studies

Table 3 presents the results of the quality assessment. The first two authors independently evaluated all included studies using the CASP criteria, then grouped and discussed their assessments. Both authors agreed that, since all studies had favourable ratings, they could be included, except for one, which required a more thorough justification for its inclusion or exclusion. To address this, further literature was consulted to deliberate on the study, i.e., Vlasova's (2017) article. However, considering the needs of the present review and Supporting Information from the literature, it was ultimately decided to include the study in this systematic review for several reasons.

1. It fulfils the inclusion criteria (such as verbal descriptions of client experiences from interviews).
2. As this was one of the few articles to focus on the experiences of traumatised asylum‐seeking refugees, it added to the variety of traumas examined in this review.
3. There is limited empirical evidence to justify excluding qualitative studies based solely on quality assessment tools (Thomas & Harden, 2008).
4. In this review, CASP was used to provide a rough overview of the characteristics of the included studies as higher‐quality articles are not void of interpretation bias.

**TABLE 3:** CASP quality assessment.

Article	1	2	3	4	5	6	7	8	9
Ashfield et al. (2021)	Yes	Yes	Yes	Yes	Yes	Yes	Yes	Yes	Yes
Bahu (2019)	Yes	Yes	Yes	Yes	Yes	Can't tell	Yes	No	Yes
Bragesjö et al. (2021)	Yes	Yes	Yes	Yes	Yes	Yes	Yes	Yes	Yes
Chouliara et al. (2020)	Yes	Yes	Yes	Yes	Can't tell	Yes	Yes	Yes	Yes
de Haan et al. (2021)	Yes	Yes	Yes	Yes	Yes	Yes	Yes	Yes	Yes
Edmond et al. (2004)	Yes	Yes	Yes	Yes	Yes	Yes	Yes	Yes	Yes
Forde and Duvvury (2021)	Yes	Yes	Yes	Yes	Yes	Yes	Yes	Yes	Yes
Gnall et al. (2020)	Yes	Yes	Yes	Yes	Yes	Can't tell	Yes	Yes	Yes
Hundt et al. (2017)	Yes	Yes	Yes	Yes	Yes	Yes	Yes	Yes	Yes
Hundt et al. (2020)	Yes	Yes	Yes	Yes	Yes	Yes	Yes	Yes	Yes
Joubert and Guse (2022)	Yes	Yes	Yes	Yes	Yes	Yes	Yes	Can't tell	Yes
Kehle‐Forbes et al. (2022)	Yes	Yes	Yes	Yes	Yes	Yes	Yes	Yes	Yes
König et al. (2020)	Yes	Yes	Yes	Yes	Yes	Yes	Yes	Yes	Yes
Lawrence and Lee (2014)	Yes	Yes	Yes	Yes	Yes	Yes	Yes	Yes	Yes
Lowe and Murray (2014)	Yes	Yes	Yes	Yes	Yes	Can't tell	Can't tell	Yes	Yes
Matheson and Weightman (2021)	Yes	Yes	Yes	Yes	Yes	Yes	Yes	Yes	Yes
May et al. (2022)	Yes	Yes	Yes	Yes	Yes	Yes	Yes	Yes	Yes
McGregor et al. (2006)	Yes	Yes	Yes	Yes	Yes	Yes	Can't tell	Yes	Yes
Middle and Kennerley (2001)	Yes	Yes	Yes	Yes	Yes	Yes	Yes	Yes	Yes
Mirdal et al. (2012)	Yes	Yes	Yes	Yes	Yes	Yes	Yes	Yes	Yes
Mott et al. (2013)	Yes	Yes	Yes	Yes	Yes	Yes	Yes	Yes	Yes
Murray et al. (2016)	Yes	Yes	Yes	Yes	Yes	Yes	Can't tell	Yes	Yes
Røberg et al. (2018)	Yes	Yes	Yes	Yes	Yes	Yes	Yes	Yes	Yes
Schwarz et al. (2020)	Yes	Yes	Yes	Yes	Yes	Yes	Yes	Yes	Yes
Schwarz et al. (2021)	Yes	Yes	Yes	Yes	Yes	Yes	Yes	Yes	Yes
Shearing et al. (2011)	Yes	Yes	Yes	Yes	Yes	Yes	Can't tell	Yes	Yes
Sherrill et al. (2022)	Yes	Yes	Yes	Yes	Yes	Yes	Can't tell	Yes	Yes
Stige, Rosenvinge, et al. (2013)	Yes	Yes	Yes	Yes	Yes	Yes	Yes	Yes	Yes
Stige, Binder, et al. (2013)	Yes	Yes	Yes	Yes	Yes	Yes	Yes	Yes	Yes
Stige et al. (2019)	Yes	Yes	Yes	Yes	Yes	Yes	Yes	Yes	Yes
Thoresen et al. (2022)	Yes	Yes	Yes	Yes	Yes	Yes	Yes	Yes	Yes
Valentine and Smith (1998)	Yes	Yes	Yes	Yes	Yes	Yes	Yes	Can't tell	Yes
Vincent et al. (2013)	Yes	Yes	Yes	Yes	Yes	Yes	Yes	Yes	Yes
Visagie and Keet (2021)	Yes	Yes	Yes	Yes	Yes	Can't tell	Yes	Can't tell	Yes
Vlasova (2017)	Can't tell	Yes	Yes	Yes	Yes	No	Can't tell	Can't tell	Yes
Wästlund et al. (2023)	Yes	Yes	Yes	Yes	Yes	Yes	Yes	Yes	Yes
Younan et al. (2018)	Yes	Yes	Yes	Yes	Yes	Yes	Yes	Yes	Yes

*Note*: 1. Was there a clear statement of the aims of the research? 2. Is a qualitative methodology appropriate? 3. Was the research design appropriate to address the aims of the research? 4. Was the recruitment strategy appropriate to the aims of the research? 5. Was the data collected in a way that addressed the research issue? 6. Has the relationship between the researcher and participants been adequately addressed? 7. Have ethical issues been taken into consideration? 8. Was the data analysis sufficiently rigorous? 9. Is there a clear statement of findings?

### Data analysis

The thematic synthesis yielded a three‐level structure for describing the results in this review. (Presented in Table 4). The first‐level themes, titled ‘descriptive subthemes’, encompass the entire content of the selected dataset. These were grouped to form the 19 second‐level themes titled ‘themes’. The third stage of the synthesis, which involves developing analytical themes, resulted in the third‐level themes titled ‘superordinate themes’. The first four superordinate themes—Intervention as a Helpful Process, Intervention as a Challenging Process, Positive Relational Aspects of the Intervention, and Negative Relational Aspects of the Intervention—addressed the first research question regarding helpful and challenging factors in the interventions. The last two, Positive Change and No Adequate Change, respond to the second research question concerning the perceived impact of the intervention. Superordinate themes, themes, and descriptive subthemes are presented in Table 4.

**TABLE 4:** Superordinate themes, themes, and subthemes identified in thematic analysis.

Superordinate themes	Themes	Descriptive subthemes
Intervention as a helpful process	Receptivity towards intervention (17)	Motivation for change
		Involvement in the intervention process
		Prioritising the intervention
	Experiencing ambivalence (21)	Scepticism towards intervention
		Demanding but helpful process
	Specific modality factors (20)	Imaginal exposure (condensed internet‐delivered Prolonged Exposure)*
		EMDR method (EMDR)*
		Cognitive restructuring (CPT)*
		Focusing on strengths (Solution‐focused Brief Therapy)*
		Emotion‐focused exercises (Schema Therapy)*
	General factors of the intervention process (32)	Psychoeducation to understand trauma reactions
		Making sense of the experience by talking and being heard
		Structure of the intervention
		Intervention as a safe place to experience emotions
		Introduction of intervention and/or exercise
		Learning specific techniques or doing exercises
		Positive external living and social factors
	Proceeding with the intervention (17)	Respectful compliance with clients' pace
		Early success in intervention helped to continue
		Intensive structure unavoidable but beneficial
Intervention as a challenging process	Challenges faced during intervention (20)	Increase in physical and emotional distress
		External living, social, and practical factors hindering intervention progress
		Dissatisfaction with intervention factors or structure
		Lack of receptivity for intervention
		Comorbidity
Positive relational aspects of the intervention	Therapeutic relationship (20)	Importance of taking time to build relation before demanding practices
		Intervention as a collaborative process
	Relationships with group (13)	Hearing others enhanced understanding
		Sharing experiences decreased sense of isolation
		Increased sense of belonging
	Therapist characteristics (25)	Trustworthy and provided sense of safety
		Understanding and empathetic support
		Non‐judgemental and accepting
		Competent
		Adapting intervention to client
		Genuine human interaction
Negative relational aspects of the intervention	Therapeutic relationship (4)	Lack of time to build relation before demanding practices
		Lack of safety to share emotions
	Relationships with group (7)	Distrust in group members
		Unable to identify with group
		Social anxiety
		Hearing others was stressful
	Therapist characteristics (5)	Not listening to clients' needs
		Unpleasant emotional expression of therapist
		Inability to listen to trauma description or misinterpreting trauma
		Mismatch of demographics
Positive change	Descriptions of change align with interventions' theories (19)	More compassionate relationship with self (Compassion‐focused therapy)*
		Changing beliefs (CPT)*
		Change at deep level (EMDR)*
		Aware of strengths (Solution‐Focused Brief Therapy)*
		Connected different parts of themselves (Schema Therapy)*
		Change in symptoms (TF‐CBT)*
	Deeper self‐understanding (15)	Realisation that trauma and consequences were not clients' fault
		Healthy view of self
	Acquisition of new life (15)	Hope for future
		Discovering opportunities in different life domains
		Positive outlook towards relationships
	Personal empowerment (16)	Greater awareness of emotional and physical condition
		Ability to understand symptoms and respond to triggers
		Accessing resources to manage emotional needs
		Increased acceptance of trauma
		Increased contact with body
		Assertiveness to express feelings and establish boundaries
	Symptoms (15)	Improvement in cognitive functioning
		Decrease in post‐traumatic symptoms
	Emotions (14)	Reduction in feelings of shame and guilt
		Decrease in negative emotions associated with traumatic event
		Emotional relief and positive change in mood and feelings
		Cognitive space for experiencing variety of emotions
No adequate change	Post‐intervention struggles (6)	Disappointment with lack of change
		Need for more intervention to resolve trauma‐related issues

* Numerical in parentheses state the frequency of repetition from 37 articles. Asterisks exhibit examples of interventions reflected across the board.

The following section provides a detailed description of the 19 second‐level themes within their respective superordinate themes. (Table 5 provides the frequencies of themes across the data.)

**TABLE 5:** Frequencies of themes per article and articles per theme.

	1	2	3	4	5	6	7	8	9	10	11	12	13	14	15	16	17	18	19	Total themes per article
Ashfield et al. (2021)	X	X		X			X	X	X				X	X	X	X	X	X		12
Bahu (2019)		X	X	X		X		X	X						X					7
Bragesjö et al. (2021)	X	X	X	X	X	X			X	X				X						9
Chouliara et al. (2020)	X			X		X		X			X									5
de Haan et al. (2021)	X	X	X	X	X		X		X					X	X	X			X	11
Edmond et al. (2004)			X	X			X		X				X	X		X		X	X	9
Forde and Duvvury (2021)	X	X		X	X													X		5
Gnall et al. (2020)			X	X		X		X			X		X	X	X	X				9
Hundt et al. (2017)	X	X	X	X	X	X	X	X	X				X	X	X	X	X	X		15
Hundt et al. (2020)			X			X				X	X	X					X		X	7
Joubert and Guse (2022)	X		X	X			X		X				X	X	X	X		X		10
Kehle‐Forbes et al. (2022)	X	X		X	X	X	X		X			X	X							9
König et al. (2020)			X	X			X		X											4
Lawrence and Lee (2014)		X		X			X	X	X				X	X	X					8
Lowe and Murray (2014)	X	X	X	X	X		X		X				X		X	X	X	X	X	13
Matheson and Weightman (2021)				X	X	X	X	X	X											6
May et al. (2022)	X	X		X	X	X	X		X	X		X				X	X			11
McGregor et al. (2006)				X	X	X	X		X	X		X								7
Middle and Kennerley (2001)				X	X		X		X			X								5
Mirdal et al. (2012)				X		X	X		X											4
Mott et al. (2013)	X	X						X			X									4
Murray et al. (2016)		X	X			X	X		X											5
Røberg et al. (2018)	X		X	X		X		X			X		X	X			X			9
Schwarz et al. (2020)			X	X									X	X	X	X	X			7
Schwarz et al. (2021)			X	X									X	X	X	X	X	X		8
Shearing et al. (2011)	X	X	X	X	X	X	X		X				X		X	X	X	X		13
Sherrill et al. (2022)	X	X		X	X	X		X	X		X		X				X			10
Stige, Rosenvinge, et al. (2013), Stige, Binder, et al. (2013) and Stige et al. (2019)	X	X*	X*	X*	X	X		X*	X		X		X^†^	X*	X*	X^†^	X	X		15
Thoresen et al. (2022)		X	X	X	X	X		X	X				X		X		X	X		11
Valentine and Smith (1998)	X	X	X	X	X		X		X				X			X	X	X		11
Vincent et al. (2013)		X		X	X	X	X		X					X	X	X	X	X	X	12
Visagie and Keet (2021)				X		X			X				X							4
Vlasova (2017)		X		X		X	X		X									X	X	7
Wästlund et al. (2023)	X	X	X	X	X			X					X	X	X	X	X	X		12
Younan et al. (2018)		X	X	X			X						X	X		X				7
Total articles per theme	17	21	20	32	17	20	20	13	25	4	7	5	19	15	15	16	15	14	6	

*Note*: X denotes a study's contribution to a theme. Stige et al., articles, *shown in two articles, † shown in three articles. 1. Receptivity towards intervention; 2. Experiencing ambivalence; 3. Specific factors of modality; 4. General factors of the intervention process; 5. Proceeding in the intervention; 6. Challenges faced during intervention; 7. Positive relational: Therapeutic relationship; 8. Positive relational: Relationships with group; 9. Positive relational: Therapist characteristics; 10. Negative relational: Therapeutic relationship; 11. Negative relational: Relationships in group; 12. Negative relational: Therapist characteristics; 13. Descriptions of change in line with interventions' theory; 14. Deeper self‐understanding; 15. Acquisition of new life; 16. Personal empowerment; 17. Symptoms; 18. Emotions; 19. Post‐intervention struggles.

### Intervention as a helpful process

The following selection of second‐level themes was analysed to be indicative of specific helpful factors that reflected insights shared by clients acknowledging their readiness for improving their reservations and scepticism regarding the efficacy and effectiveness of the intervention.

*Receptivity towards Intervention* spoke of how clients view their role in the progression of the intervention. The clients spoke of being ready to not only receive support but also be motivated as active participants with ‘a strong sense of involvement’ in the process (Lowe & Murray, 2014, p. 227). Clients underscored the need to self‐prioritise the intervention and be ready for the change. This particular theme reflected a sense of proactiveness among the clients while acknowledging the need for self‐compassion towards recognising the small, gradual changes they made through the interventions (Stige, Rosenvinge et al., 2013).

*Experiencing Ambivalence* reflected clients' scepticism towards receiving any benefit from psychotherapeutic intervention. Although clients were apprehensive, they found interventions to be ‘demanding and effective’ (Bragesjö et al., 2021, p. 5) and that ‘it's worth it in the end’ (Hundt et al., 2017, p. 52). Such insights proved to be useful in processing their traumatic experiences and working towards the completion of the intervention—the process became ‘simultaneously positive and challenging’ (Ashfield et al., 2021, p. 297).

*Specific Modality Factors* presented the particularities of interventions which clients found to be effective. For example, clients found working on intrusive and unhelpful thoughts, overthinking and being persistently focused on past events through Cognitive Processing Therapy (CPT) helpful (e.g., Hundt et al., 2017; Kehle‐Forbes et al., 2022). Similarly, clients who received Solution‐focused Brief Therapy (SFBT) singled out focusing on their strengths and building resources to gain knowledge and insight into achieving targeted behaviour (Joubert & Guse, 2022). Many clients also shared their experiences of change in understanding and processing traumatic memories after EMDR and found it to be more effective than previous experiences of therapies (e.g., Schwarz et al., 2020). Similarly, working on problematic schemas and developing adaptive ones through emotion‐focused exercises in and outside therapy sessions was particularly helpful for clients who received schema therapy (Younan et al., 2018).

*General Factors of the Intervention Process* described multiple factors which clients across the included studies predominantly found helpful regardless of the type of intervention they received. A focus of many clients was the importance of introducing the intervention, what to expect, how it was planned to proceed as well as similar introductory explanations to any worksheets and exercises. Clients also found that talking through the traumatic events and understanding the post‐traumatic consequences through psychoeducation with the therapists helped them to ‘make sense of their experiences’ (May et al., 2022, p. 7). This particular factor was related to clients' experience of ‘feel[ing] heard’ (Kehle‐Forbes et al., 2022, p. 5) by their therapist and/or among group members, and this was possible because ‘they felt safe’ (Røberg et al., 2018, p. 4). Several clients highlighted that external positive factors related to personal situations such as supportive social circles, financial security, and transportation available to visit therapists also contributed to the progression and continuation of the intervention process (e.g., Mirdal et al., 2012). Moreover, most clients were satisfied with the structure of the interventions they were receiving, especially when they could understand and learn specific techniques through repetition such as relaxation exercises and be able to utilise them outside sessions (e.g., Gnall et al., 2020; Røberg et al., 2018).

When reflecting on *Proceeding with the Intervention*, many clients appreciated and emphasised the importance of keeping with their pace, both in individual and group settings. Instead of rushing through the planned steps of the therapy or not giving the clients enough time to learn and adjust to new techniques and changes, it was largely felt that the focus of the therapy and the ‘gentle pace’ (Middle & Kennerley, 2001, p. 201) provided a sense of calm to ‘feel the emotions that c[a]me to the surface’ (Forde & Duvvury, 2021, p. 638). However, clients also acknowledged that ‘being both pushed and cared for’ (Thoresen et al., 2022, p. 7) gave a sense of accountability and ‘flexibility in the use of time’ (Lowe & Murray, 2014, p. 228). Furthermore, clients also shared that the intensity of the structure made the process unavoidable, ‘connect[ed] effort with payback’ (Sherrill et al., 2022, p. 866), and ‘helped the learning process’ (Sherrill et al., 2022, p. 868).

### Intervention as a challenging process

In contrast to helpful factors, the next second‐level theme was titled *Challenges Faced During Intervention*, which reflects challenges experienced by the participants in their ongoing psychotherapeutic interventions.

A notable number of studies showed that clients felt difficulties faced during the interventions hindered their progress. Clients described the inability to ‘speak freely about their experiences’ (May et al., 2022, p. 7) and dissatisfaction with the structure such as ‘lack of applicability’ (Gnall et al., 2020, p. 478) of the intervention programme to clients' ongoing life situations. Similarly, clients also felt that the rationale of the therapeutic intervention ‘didn't fit for [them]’ (Hundt et al., 2020, p. 417) and that they felt ‘stupid using their bodies to do strange and unfamiliar exercises’ (Stige, Rosenvinge, et al., 2013, p. 424). Furthermore, clients shared challenges in dealing with emotional concerns such as ‘physical heaviness’, ‘ongoing nightmares’ (Vincent et al., 2013, p. 586), and anticipatory anxiety with the possible increase in pre‐existing symptoms of PTSD. Moreover, clients alluded to extraneous factors such as timing, scheduling concerns, parking issues, and other logistical barriers as hindrances in continuing the intervention with interest (e.g., Gnall et al., 2020). Such concerns also negatively impacted the receptivity and motivation to adhere to the intervention.

### Positive relational aspects of the intervention

This superordinate theme reflects the collection of positive experiences of the participants in one‐on‐one therapy sessions and/or with group members and the positive characteristics of therapists.

The theme of *Therapeutic Relationship* indicated the pleasant and subsequently supportive aspects of the alliance between the therapists and clients in both individual and group settings. Numerous studies reflected clients' emphasis on ‘collaboration’ (e.g., Joubert & Guse, 2022; May et al., 2022; McGregor et al., 2006; Middle & Kennerley, 2001) with their therapist as a major factor in supporting their progress. Some clients further described ‘the therapeutic relationship as a “little safe house” and a “safety valve” for them’ (Lowe & Murray, 2014, p. 228). Furthermore, many clients emphasised factors such as the time allotted to build a safe environment with the therapist before beginning difficult tasks to have played a significant role in building a positive alliance (e.g., Shearing et al., 2011). However, the comparison of EMDR and eclectic therapy in Edmond et al. (2004) indicated that clients undergoing eclectic therapy prioritised the therapeutic relationship more than those receiving EMDR.

*Relationships with Group* reflected the positive experiences clients had while working within group‐based interventions. Many clients stated that they found the small size of the groups provided a comfortable niche, a ‘cohort [that] offers social support’ (Sherrill et al., 2022, p. 868). Clients' reflections were indicative of positive group dynamics, where they were able to feel ‘a sense of belonging, identification—or connectedness, which reduced feelings of isolation’ (Ashfield et al., 2021, p. 294). Moreover, the groups provided a sense of accountability, ‘strength, hope and inspiration for making changes and motivating participants to attend [group sessions]’ (Ashfield et al., 2021, p. 294). Respect among the group members was also considered an important factor by clients. Sharing traumatic memories and listening to others' experiences provided an atmosphere of care, being heard and support for many clients, while being aware of and respecting members' boundaries. Some clients struggled to accept compassion from others. However, group interventions aided them in building receptiveness to develop self‐compassion.

Predominantly, in positive *Therapist Characteristics* clients emphasised trust in their therapists, especially when the latter showed their support in helping the clients manage difficult emotions. Clients highlighted many therapist characteristics such as being empathetic, accepting, non‐judgemental, caring, genuinely interested in giving their time and support, active listening, and believing the clients (e.g., Joubert & Guse, 2022; Lowe & Murray, 2014; Middle & Kennerley, 2001; Mirdal et al., 2012). Another key element was therapists adhering to their clients' pace during the sessions. It was shared that therapists ‘listened closely to what they [clients] wanted in terms of pace and focus on therapy’ (McGregor et al., 2006, p. 38). Furthermore, clients shared the importance of ‘finding a therapist who was able to cope with hearing about experiences of abuse’ (McGregor et al., 2006, p. 50) as well as therapists anticipating their client's needs, providing encouragement, feedback assurances, and helpful reminders (e.g., Bragesjö et al., 2021; de Haan et al., 2021; Vincent et al., 2013). From the perspective of providing professional support, the clients also indicated that therapists' competence, sensitivity, and the ability to be flexible in dealing with trauma‐related content were also important positive factors (e.g., Bragesjö et al., 2021; Kehle‐Forbes et al., 2022; Middle & Kennerley, 2001).

### Negative relational aspects of the intervention

In contrast to the previous superordinate theme, this one reflects the negative experiences participants reported throughout their interventions, including individual therapy sessions, with group members, and the negative characteristics of therapists.

The negative *Therapeutic Relationship* included the inability to share the traumatic experiences due to a lack of safety ‘to speak freely’ (May et al., 2022, p. 7). The clients shared their dissatisfaction with the time allotted to build a therapeutic alliance before proceeding with difficult tasks (e.g., Hundt et al., 2020). Bragesjö et al. (2021) stated that from the client's perspective, the online format of delivering therapy ‘made it more difficult for the psychologist to know [client] as a person’ (p. 8), thus hindering the development of a pleasant therapeutic alliance.

Negative *Relationships with Group* reflected the unpleasant experiences clients had within group contexts, including distractions from the members (Gnall et al., 2020), social anxiety as a result of attention from group members, such as ‘performance anxiety and shame reactions’ (Røberg et al., 2018, p. 6), disclosure inhibition (Sherrill et al., 2022), and being unable to ‘identify as much with the group’ (Chouliara et al., 2020, p. 2904). Furthermore, clients were dissatisfied with the amount of time they received from the therapist and/or the time they were able to interact with other members of the group.

The negative relational factors of *Therapist Characteristics* included ‘misinterpreting meanings’ of clients' traumatic experiences (McGregor et al., 2006, p. 52), denying and/or disbelieving if the client lacks trauma memory (May et al., 2022), and inability to control their emotional response and personal issues (McGregor et al., 2006). Furthermore, clients also shared self‐reflections on worrying ‘about how their therapist really viewed them’ (Middle & Kennerley, 2001, p. 202). Similarly, they shared their discomfort in not trusting what their therapist says, e.g., ‘This is what [the therapist] is saying but does [the therapist] mean that’ (Middle & Kennerley, 2001, p. 202). Some clients also referred to feeling frustrated by the unpleasant, negative moods their therapists exhibited, such as the therapist being ‘passive, or non‐responsive’ (McGregor et al., 2006, p. 52), seeming ‘angry’ (McGregor et al., 2006, p. 54), and not listening to clients' needs (McGregor et al., 2006). Kehle‐Forbes et al. (2022) also stated that veteran clients felt therapists were rigid with protocol adherence and would refer them back to the intervention protocol ‘without tailoring the treatment’ (p. 5); this also made the clients feel they were not understood by their therapists.

### Positive change

This superordinate theme reflects participants' positive and growth‐oriented experiences after the completion of their interventions.

The *Description of Change in Line with Interventions' Theory* reflected clients reporting having experienced changes that the intervention protocol proposed. For example, clients who received compassion‐focused therapy shared that they started to gradually develop self‐compassion, especially through receiving and accepting compassion from others and acknowledging the kindness and attentiveness received from therapists. This led to a positive change in their ‘negative emotional response to the concept of developing self‐compassion’ (Lawrence & Lee, 2014, p. 501). Clients who received CPT alluded that they learned about their cognitive biases and input from different perspectives through the course of the intervention helping to restructure many of their thought processes in a positive way (e.g., Edmond et al., 2004; Hundt et al., 2020). Furthermore, Trauma‐Focused Cognitive Behavioural Therapy (TF‐CBT) and EMDR produced changes in cognitive functioning and traumatic memories (e.g., Edmond et al., 2004; Schwarz et al., 2020; Vincent et al., 2013). Similarly, through interventions such as PE, clients acknowledged that site visits helped them to fill the gaps in their memories and the physical reality of the site helped to discover information which ‘facilitated spatial orientation to memory’ (Murray et al., 2016, p. 425).

*Deeper Self‐Understanding* reflected key change clients shared, i.e., ‘being able to challenge the idea that the trauma was their fault’ (Ashfield et al., 2021, p. 295). Throughout different interventions, clients reported that not only did they begin to accept the option that the trauma happened to them rather than that they were to blame for the experience or its consequences, but this self‐understanding also helped them to create a different and more compassionate relationship with themselves (e.g., de Haan et al., 2021). This reduced self‐blame also led to an increase in their self‐awareness, remembering their strengths, and the development of a sense of personal responsibility to prioritise their well‐being (e.g., Gnall et al., 2020; Stige et al., 2019; Vincent et al., 2013).

*Acquisition of New Life* indicated the positive outlook towards life that clients experienced: a change in perspective, hope for a more prosperous future, and the confidence to venture out and engage in different life experiences. Some clients also shared that ‘a focus on the future was a new experience’ (de Haan et al., 2021, p. 9). Clients shared that seeing the change within themselves also aided their hope for continued improvement in their symptoms and concerns in different domains of life (e.g., de Haan et al., 2021; Hundt et al., 2017; Lowe & Murray, 2014). Similarly, this new feeling encouraged clients to believe in themselves, accept the trauma as part of life, and be open to new future possibilities (Joubert & Guse, 2022; Schwarz et al., 2021).

The theme of *Personal Empowerment* portrayed the increase in awareness of physical and emotional triggers, developing more connectedness with the body (e.g., Schwarz et al., 2020). Subsequently, clients also learned about and exercised techniques on how to respond to different triggers. Moreover, the increased acceptance of traumatic events led to accessing a variety of resources needed to manage psychological distress (e.g., Younan et al., 2018). Clients also stated that learning to assert personal boundaries was an empowering experience and helped to develop a more positive view of themselves (e.g., de Haan et al., 2021). Assertion of boundaries was also seen as ‘becoming an advocate for one's own needs’ (Stige, Binder, et al., 2013, p. 7) and learning to recognise and accept their limitations and needs, thus ‘letting go of excessive focus on others and increased self‐care’ (Stige, Binder, et al., 2013, p. 10).

*Symptoms* highlight clients' noticeable changes ranging from improvement in their critical thinking and executive function (such as an increase in concentration) to an evident decrease in post‐traumatic symptoms (e.g., Schwarz et al., 2021; Thoresen et al., 2022; Vincent et al., 2013). These changes also helped clients become aware of the possibility that they could help themselves to influence their symptoms (Stige, Rosenvinge et al., 2013), thus enabling them to have ‘more control over their internal and external lives’ (Schwarz et al., 2021, p. 209).

*Emotions* denote clients' mentions of changes concerning emotions. A highlight of changes in emotional experiences was a reduction in feelings of shame and guilt (e.g., Matheson & Weightman, 2021; Stige, Rosenvinge et al., 2013). Clients also reported ‘feeling more at ease with what happened to them’ (Edmond et al., 2004, p. 266), which in turn alleviated their general mood (Vincent et al., 2013) and helped in ‘gaining a sense of enjoyment from life’ (Lawrence & Lee, 2014, p. 502). Additionally, positive changes in emotions also allowed for and ‘freed up cognitive space for more emotions’ (Schwarz et al., 2021, p. 212). An increase in emotional awareness helped clients to ‘let go of a lot of [unpleasant] emotions’ (Forde & Duvvury, 2021, p. 639).

### No adequate change

The last superordinate theme reflects the participants' unpleasant experiences post‐intervention and provides one theme, *Post‐intervention Struggles*.

This theme addressed the overall dissatisfaction and disappointment of clients after interventions had concluded. Some clients felt that they did not experience a satisfactory amount of change in their life circumstances (e.g., Edmond et al., 2004) and would continue to require more psychotherapeutic interventions to resolve their traumas. This seemed to be especially true for clients with a sexual abuse history (e.g., Edmond et al., 2004). Similarly, experiencing an increase in post‐traumatic symptoms and heightened comorbidity were also factors which made clients feel disgruntled with the overall experience, especially refugee clients who felt their expectations of managing both psychological and legal matters were not resolved and/or addressed appropriately throughout the intervention (e.g., Bahu, 2019).

## DISCUSSION

This systematic review aimed to comprehensively analyse and synthesise the experiences of adult clients of psychotherapeutic interventions addressing trauma across multiple psychotherapeutic modalities. Through a thematic synthesis of 37 studies involving the experiences of a total of 1045 participants, this review sheds light on helpful and challenging factors and the impact of interventions addressing trauma from the client's perspective. The analysis revealed 19 upper‐level themes and 71 subthemes, categorised under six superordinate themes of which the first four answered the first research question of clients' experiences of the ongoing factors of the interventions: *Intervention as a helpful process*, *Intervention as a challenging process*, *Positive relational factors of the intervention*, *Negative relational factors of the intervention*, and the last two to the second research question of clients’ perceptions of the impact post‐intervention: *Positive change* and *No adequate change*. These findings highlight the diverse ways in which individual clients experience different psychotherapeutic modalities addressing trauma.

The findings of this study support insights derived from quantitative studies on the significance of common factors in psychotherapy, encompassing elements such as therapeutic alliance, empathy, and the therapist's role. In the common factors literature, therapeutic factors of interventions are delineated into specific and general factors within interventions (Wampold, 2015). Notably, clients frequently emphasised the specific factors intrinsic to particular interventions addressing trauma, aligning with Wampold and Imel's (2015) definition of ‘specific ingredients’ as helpful. Additionally, the perceived post‐intervention impacts closely corresponded with the theoretical underpinnings of the respective interventions. Beyond the specific factors, this study identified a multitude of both helpful and challenging factors often referred to as ‘general factors’ (Wampold & Imel, 2015). These factors and perceptions of post‐intervention impacts transcended the specific psychotherapeutic modalities in use. This encompassed the significance of relational factors within interventions, a theme echoed by the importance of the therapeutic alliance in individual psychotherapy and group cohesion in group therapy (Norcross & Wampold, 2011). In the current study, therapeutic relationship was one of the most mentioned factors by clients across various interventions, with studies focusing on group interventions highlighting clients' experiences that underscored the importance of relationships in group settings, fostering a sense of belonging, facilitating sharing, and providing a platform for understanding through the perspectives of others. Parry and Simpson (2016) also underscored the relational factors in their systematic review focusing on the experiences of adult survivors of child sexual abuse (CSA) in nonspecific and trauma‐focused talking therapies. In their study, healing was described as an ongoing process facilitated through trust, safety, equality, and finding connections with others. They noted that even though relational factors of therapeutic alliance are recognised as important with many client groups, survivors of CSA appeared to need specific relational experiences with the therapist, and/or experiencing connection and sharing in groups to move forward in the therapeutic process. This current review revealed that in addition to the formation of a therapeutic alliance, clients emphasised the meaningfulness of the relationship through the appraisal of specific intervention factors, such as introducing tasks and/or exercises, and especially, proceeding with their pace while adapting the intervention (especially if manualised) to their needs. Previous literature shows contrasting views regarding therapist adherence, i.e., some literature shows a significant impact on outcome (Steil et al., 2023) whereas others show no meaningful associations (Paivio et al., 2004). However, the present review specifically revealed that rigid adherence to treatment protocol made clients feel that the therapist did not know or understand them (Kehle‐Forbes et al., 2022).

The findings of this systematic review underscore the importance of involving clients in discussions and decisions regarding the various interventions available to them. They highlight the need for clients to actively participate in conversations about tailoring interventions to their individual needs both before and during the intervention process. This proactive involvement empowers clients to share insights into potential outcomes, including the possibility of heightened symptoms during the intervention. The review identified challenges clients faced such as an increase in emotional or physical distress. Previously, Whitehouse (2021) and Shipley et al. (2022) found in their systematic reviews of clients' experiences of EMDR that EMDR was talked about in a transformative manner. However, Shipley et al. (2022) also included grey literature which stated that EMDR was not a positive experience for all clients. Although quantitative literature on trauma‐focused treatments does not show symptoms exacerbating mid‐treatment (Purnell et al., 2024), the qualitative aspect of the present review adds nuanced details regarding increased distress mid‐treatment. Moreover, clients often experienced ambivalence towards the intervention, particularly during certain stages, but most clients reported eventually finding the experience beneficial. This ambivalence was particularly notable in exposure‐ or experiential‐based tasks, such as clients' reliving experiences in Cognitive Behavioural Therapy (CBT) (Shearing et al., 2011). The findings by Gjerstad et al. (2024) in their systematic review of experiences of trauma‐focused therapy for adults with PTSD were also congruent. They underscored that although many participants reported high levels of distress and considered dropping out, only a minority did, and most clients expressed that the hardships in therapy were necessary for improvement with PTSD. As the current review included studies on a variety of interventions addressing trauma (such as compassion‐focused therapy and phase‐oriented intervention), and not only trauma‐focused modalities, it reveals that ambivalence and/or challenging experiences of interventions can be experienced across the board during interventions. This adds to the literature and emphasises that facilitating discussions throughout the intervention process could play a pivotal role in enhancing clients' commitment, fostering a sense of safety, and mitigating the risk of early dropout from the intervention.

Out of the 37 studies included in this systematic review, 18 were published after 2020, signifying a notable increase in attention towards clients' experiences in psychotherapy research. This growing emphasis reflects an evolving understanding of psychotherapy and highlights the significance of delving into clients' perspectives. Numerous researchers (e.g., Levitt, Pomerville & Surace, 2016) have emphasised the crucial role of understanding clients' experiences in psychotherapy. This knowledge can affect learning about effective psychotherapeutic practices, enhancing therapists' sensitivity to clients' needs, tailoring and developing interventions, and recognising clients as active agents of change. Consequently, the expanding body of research knowledge on clients' experiences significantly contributes to advancing these critical objectives.

## LIMITATIONS AND STRENGTHS

This review entails certain limitations that warrant acknowledgement. Primarily, the analysis was predominantly conducted by the first author, although regular discussions were held with the second and other authors. This single‐author involvement introduces a potential risk of bias. Furthermore, as is characteristic of qualitative research methods, it is important to recognise that different researchers may produce diverse findings. In addition, the majority of the original studies did not provide information on the expertise or years of experience of the therapists involved and a significant portion of the included intervention modalities were brief interventions. The effect of therapists' experience or longer interventions could not be accounted for. Notably, only one study reported client experiences one year after the intervention, while others presented findings immediately following the conclusion of the intervention. This raises questions about the long‐term effects of these interventions. Furthermore, it is important to note that this study specifically focused on conceptualising challenging phenomena, in the context of interventions explicitly targeting the consequences of trauma, such as PTSD/CPTSD, and did not include trauma treatment where it is not the focus of the treatment.

This systematic review boasts two important strengths. First, the inclusion of studies spanning a diverse range of psychotherapeutic intervention modalities (excluding for example psychotherapeutic somatic approaches for which no studies with this systematic review's eligibility criteria were found) suggests that the findings may hold relevance and applicability across a wide spectrum of interventions. This broad inclusion enhances the potential transferability of the study's conclusions. Secondly, the review's sample size is notably large for a qualitative systematic review including a range of participants across different age groups, ethnicities, and different types of traumas. This substantial sample facilitates a broad yet comprehensive understanding of the phenomenon under investigation, and future studies could aim at an in‐depth understanding of the various factors impacting interventions identified through this systematic review.

## AUTHOR CONTRIBUTIONS

**R. Lepistö:** Conzeptualization; Methodology; Investigation; Writing – original draft; Formal analysis; Validation; Visualization. **A. Ahmad:** Conzeptualization; Methodology; Validation; Investigation; Writing – Original draft; Visualization. **S. Kangaslampi:** Conzeptualization; Methodology; Writing – Review & editing; Supervision. **K. Peltonen:** Conzeptualization; Methodology; Writing – Review & Editing; Supervision.

## CONFLICT OF INTEREST STATEMENT

The authors declare that there are no conflicts of interest to report.

## Supporting information


Supplementary 1.

## Data Availability

All data generated or analysed during this study are included in this article and its Supplementary Material files.
