# Supplementary material for: Clients' experiences of psychotherapeutic interventions addressing trauma
Source: Psychol Psychother. 2025 Jan 3;98(1):149–74. doi: 10.1111/papt.12569 (PMC11823356; doi:10.1111/papt.12569)
Supplement: Supplementary file 1 — Supplementary 1. [file PAPT-98-149-s001.docx]

**Supplementary 1**

*Example of search in PsycINFO*

|  | **Searches** | **Results** | **Type** |
| --- | --- | --- | --- |
| 1 | (trauma* or ptsd or ptss or post-traumatic stress*) ti,ab | 135730 | Advanced |
| 2 | (psychological treatment* or therapy* or psychotherapy* or intervention* or counseling* or behavioral* or brief eclectic* or client-centered* or cognitive* or cognitive-analytic* or cognitive processing* or dialectical behavioral* or ego-state* or emdr or eye movement desensitization and reprocessing* or emotion-focused* or feminist therapy* or gestalt* or humanistic* or hypno* or integrative* or internal family systems* or interpersonal* or narrative exposure therapy* or person-centered* or prolonged exposure* or psychoanalytic* or psychoanalysis* or psychodynamic* or sensorimotor* or solution-focused* or somatic experiencing* or trauma-focused cognitive behavioral therapy* or tf-cbt) ti,ab | 276470 | Advanced |
| 3 | (case stud* or consensual qualitative* or content analy* or discourse analysis* or ethnographic* or field study* or focus group* or grounded theory* or interview* or lived experience* or narrative* or observ* or phenomenological* or process evalua* or significant moments* or survey* or thematic analy*) ti,ab | 1283571 | Advanced |
| 4 | (attitude* or experienc* or opinion* or perce* or response* or view*) ti, ab | 1545189 | Advanced |
| 5 | (qualitative* or mixed-meth* or mixed meth* or multi-meth* or multi meth*) ti, ab | 225478 | Advanced |
| 6 | 1 and 2 | 17355 | Advanced |
| 7 | 3 or 4 or 5 | 2399791 | Advanced |
| 8 | 6 and 7 | 8191 | Advanced |
| 9 | clients/ or client attitudes/ or client satisfaction/ | 34863 | Advanced |
| 10 | trauma/ or emotional trauma/ or posttraumatic stress/ or traumatic experiences/ or cognitive processing therapy/ or posttraumatic stress disorder/ or prolonged exposure therapy/ or trauma treatment/ | 71715 | Advanced |
| 11 | 9 and 10 | 515 | Advanced |
| 12 | 8 or 11 | 8542 | Advanced |
| 13 | limit 12 to (1600 qualitative study and adulthood <18+years> and “300 adulthood <age 18 yrs and older>” and (“0110 peer-reviewed journal or “0400 dissertation abstract”) and (English or German) and human) | 627 | Advanced |
